# Effect of solid state fermentation on proximate composition, antinutritional factors and in vitro protein digestibility of maize flour

**DOI:** 10.1002/fsn3.2599

**Published:** 2021-09-22

**Authors:** Zemenu K. Terefe, Mary N. Omwamba, John M. Nduko

**Affiliations:** ^1^ Dairy and Food Science and Technology Egerton University ‐ Njoro Campus Njoro Kenya; ^2^ Food Science and Technology Hawassa University Hawassa Ethiopia

**Keywords:** antinutritional factors, in vitro protein digestibility, *Lactobacillus plantarum*, proximate composition, *Saccharomyces cerevisiae*

## Abstract

Cereals including maize generally have limiting amino acids particularly lysine. In most cases, spontaneous fermentation is used to improve the nutritional profiles of maize‐based products. However, in such fermentation, biological risks including the presence of pathogenic microorganisms, chemical contaminants, and toxic compounds of microbial origin such as mycotoxins pose a health risk. The aim of this study was, therefore, to improve the nutritional properties of maize flour by reducing antinutritional factors through microbial fermentation by strains of *Lactobacillus plantarum* and *Saccharomyces cerevisiae* and their cocultures. A factorial experimental design was used to evaluate the effect of fermentation setups and time on proximate composition, antinutritional factors, and in vitro digestibility of proteins in maize flour. During 48 h of fermentation, protein content was improved by 38%, 55%, 49%, and 48%, whereas in vitro protein digestibility improved by 31%, 40%, 36%, and 34% for natural, *Lactobacillus plantarum*, *Saccharomyces cerevisiae,* and their coculture‐fermented maize flour, respectively. The highest improvement in protein content and its digestibility was observed for *Lactobacillus plantarum* strain‐fermented maize flour. Phytate, tannin and trypsin inhibitor activity were reduced significantly (*p* < .05) for natural, *Lactobacillus plantarum*, *Saccharomyces cerevisiae,* and coculture‐fermented maize flour. The highest reduction of phytate (66%), tannin (75%), and trypsin inhibitor (64%) was observed for coculture‐fermented maize flour. The two strains and their cocultures were found feasible for fermentation of maize flour to improve its nutritional profiles more than the conventional fermentation process.

## Graphical abstract

This research showed that, fermentation with *L. plantarum*, *S. cerevisiae* and their co‐cultures resulted in an improved nutritional value of maize flour. Fermentation with these strains significantly increases the contents of protein and its digestibility of maize flour.

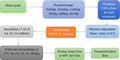

## 1 INTRODUCTION

Maize (*Zea mays* L.) is the most produced cereal crop in the world and a major source of calories for most of the world population. It accounts for 40% of the total cereal production in sub‐Saharan Africa and provides about 30% of the total calories intake of more than 4.5 billion people in developing countries (Chaves‐López et al., 2020; Michel‐Michel et al., 2020). However, the nutritional profile of maize is inferior compared to other cereal crops, especially maize is low in the essential amino acid, lysine. The over‐reliance on starch‐dense staples such as maize in sub‐Saharan Africa results in widespread dietary micronutrient deficiency and protein energy malnutrition (PEM). Due to this, a number of processing techniques have been applied on maize‐based products to ameliorate the nutritional qualities of maize based products. These processing methods, however, have their own limitations with regard to nutritional profile enhancement and antinutritional factors reduction. Nowadays, in the evolving functional food era, new sophisticated technological tools are leading to significant transformations in the field of food and nutrition (Tsafrakidou et al., 2020).

Fermentation technology is one of the forefront tools in food technology since it provides a solid foundation for the development of safe food products with better nutritional and functional attributes. It is recognized as a natural way to preserve and safeguard foods and beverages, enhancing the nutritional value, improving the digestibility and reducing antinutritional factors. A number of researches have been carried out with regard to fermentation to get fermented maize‐based products in the past couple of years (Amankwah et al., 2009; Anaemene & Fadupin, 2020; Asiedu et al., 1993; Cui et al., 2012; Ejigui et al., 2005; Forsido et al., 2020; Irtwange & Achimba, 2009; Ogodo et al., 2017, 2018). However, most of these studies are merely focused on spontaneous fermentation processes even though microbial fermentation such as use of lactic acid bacteria is more efficient in improving the nutritional profile of foods and promoting human health beneficial properties.

Microbial fermentation, especially lactic acid bacteria, have been used extensively for a variety of food products as they are confirmed as generally regarded as safe (GRAS) (Petrova, 2020). Some strains of lactic acid bacteria, mainly *Lactobacilli*, inhabit the gastrointestinal tract (GIT) of humans possessing probiotic effects, in addition to making the food easily digestible, decreasing the level of high‐chain carbohydrates and some indigestible poly‐ and oligosaccharides (Turpin et al., 2011). It has been also explained that the combination of lactic acid bacteria and yeasts in the fermentation of sourdough improves the nutritional properties and increases the volume of the subsequent bread, and softens its texture significantly (Katina & Poutanen, 2013). However, there is little or no report regarding use of pure strains of *Lactobacillus plantarum*, *Saccharomyces cerevisiae* and their cocultures for fermentation of maize flour. Hence, in this study, these two strains were evaluated with regard to their effects on proximate, antinutritional factors, and in vitro protein digestibility of maize flour during fermentation.

## 2 MATERIALS AND METHODS

### 2.1 Sample collection and preparation

Maize grain (BH 543) variety was collected from Hawassa Agricultural Research Centre, Southern Ethiopia. The grains were sorted and cleaned to remove foreign matter. Then the grains were washed with distilled water and dried in an oven (Binder) at 70°C for 7 h (Ogodo et al., 2018). The dried kernels were milled into flour using a laboratory disk miller (Alvan Blanch). The flour was sieved using 100‐µm mesh size and packed in polyethylene bag and stored in a desiccator until the fermentation process was carried out. Starter cultures; *Lactobacillus plantarum* (Lp) and *Saccharomyces cerevisiae* (Sc), previously isolated from fermented maize flour dough, were collected from Ethiopian Biodiversity Institute (EBI), Addis Ababa, Ethiopia with a 5‐ml plastic vial container and transported to the experimental site with an ice box.

### 2.2 Inoculum preparation

*Lactobacillus plantarum* (Lp) inoculum was developed following the method of Ogodo et al. (2017) with slight modifications. A standard culture of *L. plantarum* inoculum was prepared on MRS agar (Becton, Dickinson and Co.) from stock cultures frozen in MRS broth, from which isolated colonies were selected for further propagation. The *L. plantarum*bacterium in the frozen cultures was first activated in MRS broth by adding 0.1 ml of frozen culture to test tube which contained 9 ml of MRS broth and incubated for 48 h at 37°C. Then, one loopful was taken and spread‐plated on De Man, Rogosa and Sharpe (MRS) agar, after which incubation was done anaerobically at 37°C for 48 h. The cells were harvested by centrifugation at 5000 gravity for 10 min and washed with distilled water. Inoculum development of *Saccharomyces cerevisiae* (Sc) was performed according to Vilela et al. (2020) with slight modification. A stock culture of *S. cerevisiae* was activated by inoculating the cells into a freshly prepared yeast extract peptone dextrose (YPD) broth containing 2% glucose, 2% peptone and 1% yeast extract. The culture was incubated overnight at 37°C to achieve a significant growth of population, and one loopful was taken and spread‐plated on YPD agar and incubated at 30°C for 3 days.

### 2.3 Fermentation of maize flour

**FIGURE 1:**
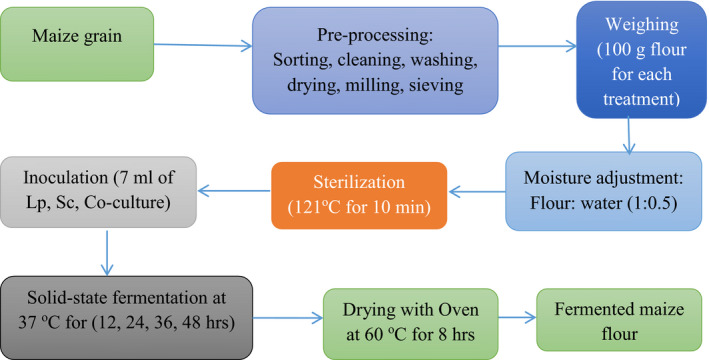
Schematic presentation of maize flour sample preparation for fermentation and analysis

The maize flour sample was fermented with four fermentation setups according to the method described by Ogodo et al. (2018) with slight modifications. The flours were mixed with distilled water in the ratio of 1:0.5 (w/v) in 500‐ml beaker and mixed thoroughly with a hand mixer. The samples were then sterilized in an autoclave at 121°C for 10 min to minimize the risk of contamination and allowed to cool for 30 min at room temperature (25 ± 2°C). The samples were then inoculated with 7 ml of 1 × 10^6^ cells/ml of *L. plantarum* and *S. cerevisiae* strains each and 3.5 ml of 1 × 10^6^ cells/ml each for cocultures and allowed to ferment in a solid state fermentation type. The beakers were covered by aluminum foils. The natural fermentation process was prepared using the same procedure without sterilization and addition of starter cultures. All the fermentation processes were carried out within an incubator (Wagtech) set at 37°C for 48 h. Samples were withdrawn at 12‐h interval for analyses. Before analysis, the fermented maize flour samples were dried in oven (Binder) at 60°C for 8 h. The overall flow of sample preparation for analysis in this research was, as indicated in Figure 1.

### 2.4 Proximate composition analysis

All proximate composition parameters were determined using AOAC (2005) methods. The moisture content was determined by drying in an oven at 105°C until constant weight was reached (Method 925.09). Crude protein was done by microKjeldhl method with an acid (sulfuric acid) digestion of the sample and then an alkaline (sodium hydroxide) distillation and nitrogen to protein conversion factor of 6.25 used (Method 979.09). Crude fat was determined using hexane extraction in a Soxhlet extraction system (Method 920.39). Crude fiber was determined as the combustible and insoluble organic residue obtained after the sample was subjected to acid (H_2_SO_4_) digestion and then alkaline (NaOH) distillation (Method 962.09). Ash content was quantified as the inorganic residue remaining after incineration of the sample at 550°C until loss of organic matter (Method 923.03). Carbohydrate content was estimated by difference (Ojokohet al., 2020).

### 2.5 Antinutrients analysis

Phytate and trypsin inhibitor in maize flour were determined according to Ogodo et al. (2018) with slight modifications. Phytate in the sample was determined using UV–VIS Spectrophotometer. The quantity of phytic acid was measured using an absorbance of molybdenum blue at 655 nm. Trypsin enzymatic activity was assayed using casein as substrate and inhibition of the activity was measured in the extract. Then absorbance metrics was plotted against the volume of extract. Trypsin inhibitor activity was then measured as the number of trypsin units inhibited (TIU). The amount of tannin in the sample was determined as percentage of catechin equivalents (% CE) according to Onyango et al. (2013).

### 2.6 Determination of in vitro protein digestibility

The method described by Galal et al. (2013) was used for determination of in vitro protein digestibility of maize flour with slight modifications. Exactly 0.2 g maize flour was placed in a 50‐ml centrifuge tube and incubated with 1.5 mg of pepsin in 15 ml of 0.1 N HCl at 37°C for 3 h and neutralized with 7.5 ml of 0.2 M NaOH. This was followed by addition of 4 mg pancreatin in 7.5 ml phosphate buffer and incubated at 37°C for 24 h. Then, 5 mg trichloroacetic acid was added to stop the reaction and centrifuged at 5000 *g* for 10 min. The mixture was filtered through Whatman No. 1 filter paper. The supernatant was dried at 50°C and followed by assaying for nitrogen using microKjeldahl method, and the in vitro protein digestibility was calculated using the following formula.

$$\text{In}\;\text{vitro}\;\text{protein}\;\text{digestibility}\;\left(\%\right)=\frac{X‐Y}{X}\times 100.$$

where X is percentage of protein in the sample before digestion and Y is percentage of protein in the sample after enzymatic digestion.

### 2.7 Experimental design and data analysis

A 4 × 5 factorial experimental design was used to evaluate the effect of fermentation types (4 levels) and time (5 levels) on proximate composition, antinutritional factors, and in vitro protein digestibility of maize flour (Table 1). All measurements were done in triplicate. Data analysis was carried out using SAS JMP pro13.0 (Richard Boulton). The data obtained were analyzed for mean differences with analysis of variance (ANOVA) using Tukey's honest significant difference (HSD) test at 5% level of significance.

**TABLE 1:** Factors (2) with levels combination

Fermentation setups	Fermentation time
Natural	0, 12, 24, 36, and 48 h
*Lactobacillus plantarum* inoculum	0, 12, 24, 36, and 48 h
*Saccharomyces cerevisiae* inoculum	0, 12, 24, 36, and 48 h
Cocultures (*L. plantarum* and *S. cerevisiae*)	0, 12, 24, 36, and 48 h

## 3 RESULTS AND DISCUSSION

### 3.1 Results

#### 3.1.1 Proximate composition

**FIGURE 2:**
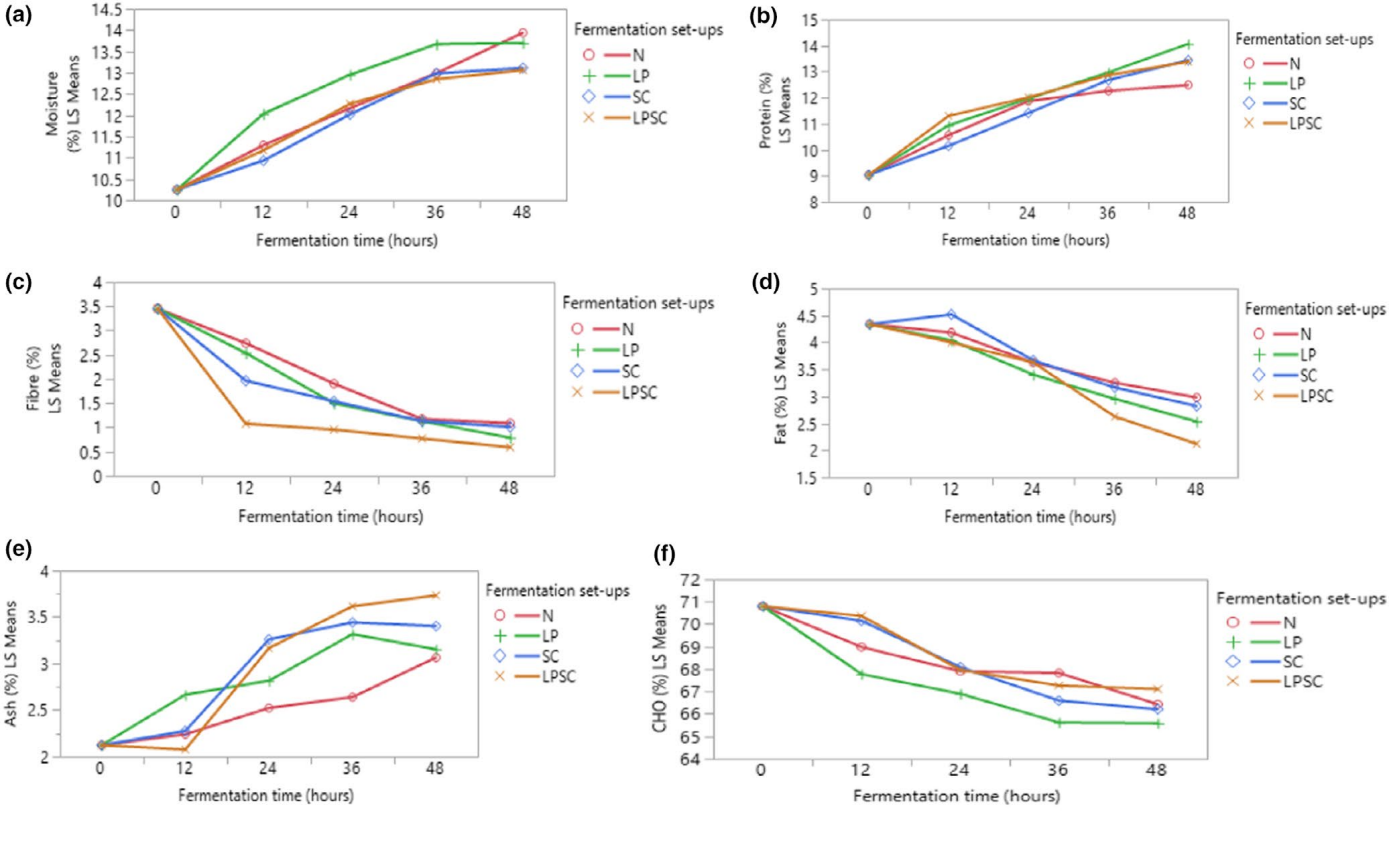
Interaction plots (fitted means) of proximate composition of maize flours fermented for up to 48 h. Moisture (a); Protein (b); Fiber (c); Fat (d); Ash (e) and Carbohydrate (CHO) (f) contents. LP, *Lactobacillus plantarum*; LPSC, mixed co‐culture of LP and SC; N, natural fermentation; SC, *Saccharomyces cerevisiae*

The results for proximate composition of maize flour fermented for 48 h are presented in Figure 2 as dry weight basis. The moisture content of maize flour increased significantly (*p* < .05) from 10.25% of the unfermented maize flour to 13.94%, 13.70%, 13.11%, and 13.06% for natural, *L. plantarum*, *S. cerevisiae*, and coculture‐fermented maize flours, respectively, at 48 h of fermentation (Figure 2a). The moisture content was found to increase with an increase in the fermentation time. The increase in moisture content of naturally fermented flour was linear compared to fermented flour by the two strains and their cocultures. However, *L. plantarum*, *S. cerevisiae*, and their cocultures inoculated fermented flour showed low increments after 36 h of fermentation time. In all the fermentation setups, protein content of maize flour significantly (*p* < .05) increased from 9.03% to 12.49%, 14.06%, 13.44%, and 13.38% for natural, *L. plantarum*, *S. cerevisiae*, and their coculture‐fermented maize flours, respectively (Figure 2b). The highest increase in protein content was observed for *L. plantarum* strain‐fermented maize flour, while the lowest was with natural fermentation. In the present study, fiber contents decreased significantly (*p* < .05) from 3.45% to 1.09%, 0.79%, 1.01%, and 0.59% for natural, *L. plantarum*, *S. cerevisiae* and their coculture‐fermented maize flour, respectively (Figure 2c). The highest decrease in fiber content was observed for coculture‐fermented maize flour, while the lowest decrease was observed with the natural fermentation. Fat contents decreased significantly (*p* < .05) from 4.34% to 2.98%, 2.54%, 2.82%, and 2.12% for natural, *L. plantarum*, *S. cerevisiae* and their coculture‐fermented flour, respectively (Figure 2d). The highest decrease in fat content was observed for coculture‐fermented maize flour while the lowest was with natural fermentation. Ash contents in the present study showed a slight increment from 2.12% to 3.07%, 3.15%, 3.40%, and 3.73% for natural, *L. plantarum*, *S. cerevisiae* and their coculture‐fermented flour, respectively (Figure 2e). However, the ash content decreased after 36 h of fermentation during *L. plantarum* and *S. cerevisiae* strains inoculated fermentation conditions. Carbohydrate content decreased significantly (*p* < .05) from 70.80% to 66.43%, 65.59%, 66.21%, and 67.11% for natural, *L. plantarum*, *S. cerevisiae*, and their coculture‐fermented flour, respectively (Figure 2f).

#### 3.1.2 Antinutritional factors

**FIGURE 3:**
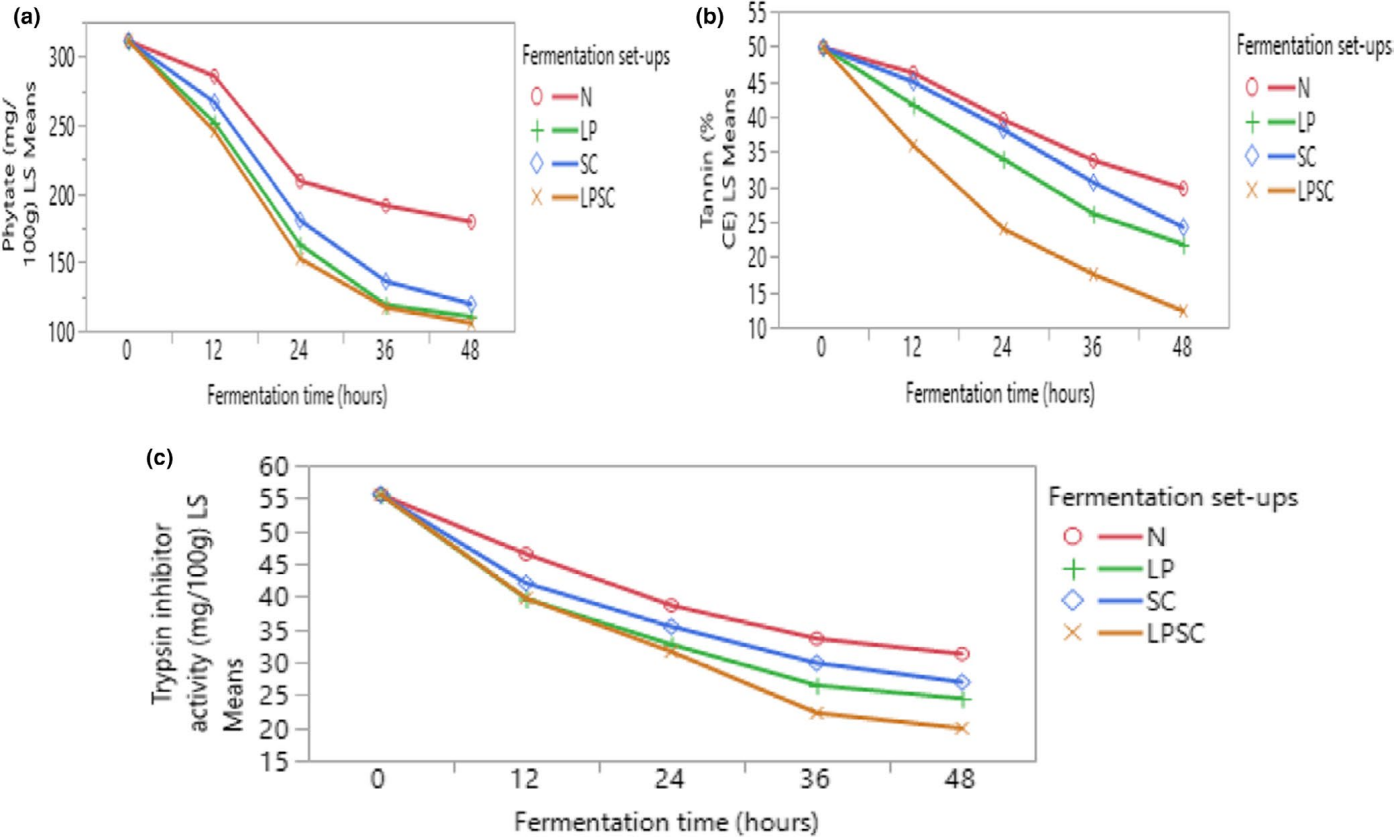
Interaction plots (fitted means) of anti‐nutritional factors of fermented maize flour for 48 h. Phytate (a); tannin (b) and trypsin inhibitor (c) contents

Phytate, tannin, and trypsin inhibitor contents decreased significantly (*p* < .05) with an increasing fermentation time in all fermentation setups (Figure 3). Phytate content decreased from 311.35 mg/100 g flour at the start of fermentation to 179.41 mg/100 g, 110.25 mg/100 g, 119.38 mg/100 g, and 105.52 mg/100 g for natural, *L. plantarum*, *S. cerevisiae*, and their coculture‐fermented flour, respectively, at 48 h of fermentation (Figure 3a). The tannin content decreased from 49.84% CE at the start of fermentation to 12.3% CE for the mixed cocultures that had the highest decrease at 48 h of fermentation (Figure 3b). Trypsin inhibitor content decreased as well from 55.56 mg/100 g at the start of fermentation to 19.94 mg/100 g at 48 h of fermentation with the mixed coculture that had the highest decrease (Figure 3c).

#### 3.1.3 In vitro protein digestibility

**FIGURE 4:**
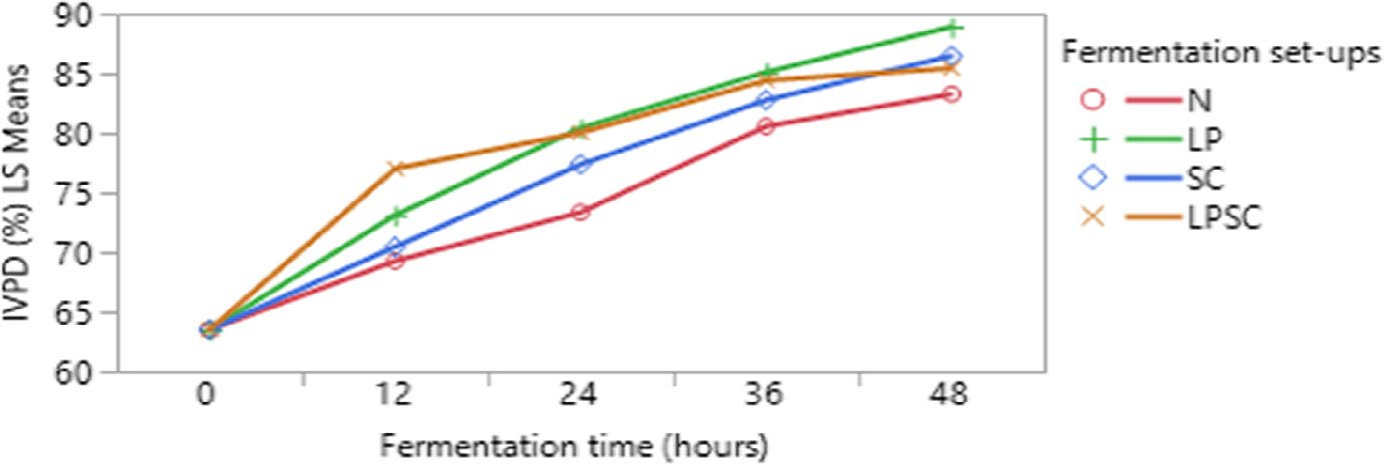
Interaction plots (fitted means) of in vitro protein digestibility of maize flour fermented for 48 h

The in vitro protein digestibility values increased significantly (*p* < .05) with an increase in fermentation time (Figure 4). The highest value (88.91%) was observed for *L. plantarum* strain‐fermented maize flour at 48 h of fermentation, while the lowest (83.26%) was obtained with natural fermentation at 48 h.

### 3.2 Discussion

Maize and its derived fermented products are fundamental for human nutrition for a great proportion of the global population (Chaves‐López et al., 2020). In fermentation, mixed cultures of lactic acid bacteria, yeasts, and sometimes molds are involved in transforming the food thereby improving its nutritional value and shelf life. In most cases, spontaneous fermentation is used for the production of maize‐fermented products. However, in such fermentation, biological risks including the presence of pathogenic microorganisms, chemical contaminants, and toxic compounds of microbial origin such as mycotoxins, biogenic amines, and cyanogenic glycosides pose a health risk (Capozzi et al., 2020). For this reason, it is important to understand the role of different microorganisms in fermenting maize to optimize the final quality, improve food safety of the products, and understand their effect on the nutritional composition.

In this study, maize flour was fermented using *Lactobacillus plantarum*, *Saccharomyces cerevisiae*, cocultures of both organisms and spontaneous fermentation was used as a control. The effect of fermentation on the nutritional composition of maize flour was evaluated over a period of 48 h. The moisture content of naturally fermented maize flour in the present study (13.94%) was higher than 10.82% obtained by Ogodo et al. (2017) but less than 14.2% reported by Assohoun et al. (2013). However, the values of moisture content of naturally fermented maize flour in the present study was in agreement with that obtained by Mbata et al. (2009), who reported 13.20% moisture content in fermented maize flour. The moisture content of lactic acid bacteria consortium including *L. plantarum‐*fermented maize flour increased from 9.66% to 10.82%, similar to that reported by Ogodo et al. (2017) which is less than 12.03% to 13.94% increment of the present finding. The increase in moisture content could be attributed to the addition of a calculated amount of water to the substrate prior to solid state fermentation. Moreover, during natural fermentation, a consortium of microbial strains initiates the fermentation process to continue for long period of time, and this could lead to the moisture content of fermented maize flour to increase throughout the fermentation period (Sharma et al., 2020). However, during *L. plantarum*, *S. cerevisiae* and their cocultures inoculated fermentation process, uninterrupted increase in moisture content continued only up to 36 h of fermentation. This might be due to the fact that microorganisms actively utilizes the substrate during logarithmic growth until the nutrients are depleted and produces products such as lactic acid, acetic acid including water depending on the ingredients used for fermentation (Sharma et al., 2020).

The protein content of maize flour naturally fermented for 48 h (12.49%) in the present study was higher than 10.44% reported by Anaemene and Fadupin (2020) but lower than 18.4% reported by Mbata et al. (2009). The protein content (14.06%) of *L. plantarum* strain‐fermented maize flour in the present study is higher than 12.97% reported by Ogodo et al. (2017). After 48 h of fermentation, the increase in protein content in *L. plantarum*‐fermented maize flour was higher than that of natural fermentation, *S. cerevisiae* strain, and coculture‐fermented maize flours. This could be due to the ability of lactic acid bacteria like *L. plantarum* to secrete some extracellular enzymes which are proteins (Oseni & Akindahunsi, 2011). It was also reported that *L. plantarum* can produce different enzymes and biomolecules, which are proteinaceous in nature during fermentation, hence increasing the protein content of the products (Siezen et al., 2011). The protein content (13.44%) of *S. cerevisiae* strain‐fermented flour in the present study is in agreement with that of Banik et al. (2020), who reported 13.68% of protein for multigrain‐based food after fermentation (4 days) by *S. cerevisiae*. The increase in the protein contents of naturally fermented maize flour with an increasing fermentation time could be attributed to the logarithmic growth of different strains of microorganisms during fermentation, which produces proteolytic enzymes, increasing the protein content (Ojokoh et al., 2020). Moreover, the increase in protein content of maize flour after fermentation could also be attributed to a decrease in carbon ratio in the total mass and an increase in cell biomass and productions of nonprotein nitrogen compounds like ammonia, amines, amino acids, and peptides as these all are included in the crude protein content (Onyango et al., 2013). During fermentation, microorganisms utilize carbohydrates as an energy source and produce carbon dioxide as a by‐product and this causes the nitrogen in the fermented product to be concentrated, and thus, the proportion of protein in the total mass increases (Nasseri et al., 2011).

The fiber content of naturally fermented maize flour in the present study decreased from 3.45% to 1.09% which is in agreement with the finding of Anaemene & Fadupin, 2020 who reported a crude fiber content of 1.18% in maize flour after 72 h of fermentation. However, the fiber content in the present study is much lower than 5.20% reported by Mbata et al. (2009). More decrease in the fiber content (3.45% to 0.59%) was observed for coculture‐fermented maize flour. The trend in fiber content decrease in the present study is similar with the reported values of fiber content of lactic acid bacteria consortium‐fermented maize flour by Ogodo et al. (2017). The decrease in the crude fiber content of fermented maize flour in the present study could be attributed to the secretion of extracellular enzymes by microorganisms that hydrolyze and metabolize insoluble polysaccharides. It has been also reported that the enzyme ß‐D‐glucosidase is produced by bacteria such as *L. plantarum* and this enzyme is able to hydrolyze terminal, nonreducing part of the polysaccharide chains (Minnaar et al., 2017). The fermentation process usually decreases the soluble dietary fiber more than the insoluble dietary fiber content and this makes total crude fiber content of the fermented products to decrease (Brennan et al., 2013; Bunzel et al., 2001; Comino et al., 2018). The decrease in fiber contents after fermentation is an indication of softening of fibrous tissues and increased digestibility due to activities of microorganisms which are known for the bioconversion of carbohydrates and lignocellulose into protein (Adegunloye & Oparinde, 2017; Igbabul et al., 2014).

The fat content of naturally fermented maize flour decreased from 4.34% to 2.98% in the present study. The value of this decrease in the present study is lower than 5.2% to 3.76% reported in other studies (Amankwah et al., 2009; Gernah et al., 2011; Ogodo et al., 2017). This might be due to the initial fat composition of maize grain as the fat content varies with variety and growing conditions. However, the present finding is in consistent with the reported value of 2.77% by Opeifa et al. (2015) and 2.48% by Irtwange and Achimba (2009). The fat content decreased to 2.54%, 2.82% and 2.12% for *L. plantarum*, *S. cerevisiae* strains and their coculture‐fermented maize flour, respectively. The decrease in fat content by cocultures was more than that with *L. plantarum*, *S. cerevisiae* strains and naturally fermented maize flour. The value of fat decrease in the present study was lower than the 4.08% reported by Ogodo et al. (2017) for LAB consortium‐fermented maize flour after 48 h of fermentation. The decrease in fat content might be attributed to the utilization of fat for energy source by microorganisms for their metabolic activities during fermentation. Moreover, the reduction in fat content might be as a result of the oxidation process that could happen during fermentation (Fasasi et al., 2007; Li et al., 2020).

The ash content showed a slight increment from 2.12% to 3.73% in the present study. However, the difference in ash content across the four types of fermentation medium was not significant (*p* > .05) except for 36 and 48 h of fermentation time. Similar trend was observed by Ogodo et al. (2017) and Oluwamukomi et al. (2005) who reported an increment in ash content of fermented maize flour from 1.88% to 3.14% and 2.37% to 2.75%, respectively. The highest increment (3.73%) of ash content in the present study was observed for cocultures followed by *Saccharomyces cerevisiae* strain (3.40%) fermented flour. The slight increment in ash content could be attributed to loss of organic matter and accumulation of inorganic matter caused by the activities of enzymes and microorganism during fermentation (Uvere et al., 2010).

The highest decrease (65.59%) in carbohydrate content of fermented maize flour in the present study was observed for *L. plantarum* strain‐fermented maize flourwhereas, the lowest decrease (67.11%) was observed for coculture‐fermented maize flour. The current finding is in agreement with the finding of Ogodo et al. (2017) who reported 70.82% to 68.01% decrease in carbohydrate content of LAB consortium‐fermented maize flour. Similar trend was reported by Ojokoh et al. (2014) who found 74.2% to 66.66% decrease in carbohydrate content of bread fruit and cowpea blended fermented flour, respectively. The change in carbohydrate content might be due to the increasing or decreasing values of other chemical compositions like moisture, protein, fiber, fat, and ash by the effect of fermentation process since the carbohydrate content was determined based on difference method. Moreover, the decrease in carbohydrate content could be attributed to the use of carbohydrate as a source of energy by microorganisms during fermentation (Nasseri et al., 2011).

Antinutritional factors are the major limiting components in cereals for nutritional bioavailability and they aggravate nutrition related problems in humans. In the present study, after 48 h of fermentation of maize flour with four fermentation setups, the contents of phytate, tannin, and trypsin inhibitor activity were reduced significantly (*p* < .05). The phytate content in the fermented maize flour was reduced from 311.35 mg/100 g to 105.52 mg/100 g. The higher reduction in phytate was observed for cocultures followed by *L. platurum* strain, *S. cerevisiae* strain, and naturally fermented flour. This could be due to the fact that these microorganisms are the source of phytase enzyme which can degrade phytate (Handa et al., 2020; Sandberg & Andlid, 2002). The amount of phytate reduction in the present study is higher than Ogodo et al., (2018), who reported a reduction from 296.10 mg/100 g to 76.76 mg/100 g for LAB consortium‐fermented maize flour. This might be due to the difference in the initial contents of ingredient and the fermentation setups used in the present study. Phytic acid is the major storage form for phosphorus in the cereal grains and exists in the form of mixed salts of Ca–Mg–K (phytate) and occurs in many locations within the kernel (Wu et al., 2009). It forms complexes with dietary minerals, and causes mineral‐related deficiency in humans and it also negatively affects protein and lipid utilization (Coulibaly et al., 2010; Kumar et al., 2010). The reduction in phytate which is found in the form of *myo*‐inositol hexa‐phosphate (IP_6_) in cereals, could be attributed to production of phytase enzyme during the fermentation process that facilitates the degradation process (Selle et al., 2000; Troesch et al., 2013). Phytase can be produced naturally (endogenous phytase) or by microorganisms (exogenous phytase). Optimal temperature for phytase activity has been known to range between 35°C and 45°C (Sindhu & Khetarpaul, 2001). In the present study, the temperature used during fermentation was 37°C which favors effective phytate reduction process by phytase enzyme. Phytases have the capacity to dephosphorylate phytate in a step‐wise manner to a series of lower inositol phosphate esters (myo‐inositol penta‐phosphate to myo‐inositol mono‐phosphate) and ultimately, to inositol and inorganic phosphorus (Selle et al., 2000). This enzyme breaks down the phosphate bond and further reduces the most reactive inositol hexa‐phosphate into the least reactive inositol mono‐phosphate. Therefore, de‐phosphorylation of phytate is a prerequisite for improving nutritional value of foods with various processing methods because removal of phosphate groups from the inositol ring decreases the mineral binding strength of phytate.

In the present study, the tannin content was reduced significantly (*p* < .05) from 49.84% CE to 12.3% CE. The highest reduction was observed for cocultures followed by *L. platurum* strain, *S. cerevisiae* strain, and naturally fermented flour. This finding is in agreement with Ogodo et al., (2018) who reported a reduction of 43.64% to 31.38% for LAB consortium‐fermented maize flour. Tannins are a chemically diverse group of water soluble phenolic compound which binds proteins to form soluble or insoluble complexes and alter their structural and functional properties (Girard et al., 2018). The dietary proteins form complexes with phenolic compounds via noncovalent or covalent interactions. Both reaction mechanisms could affect the chemical structures of interacted proteins and phenolics, thereby inducing changes in their nutritional, functional and biological characteristics, as well as product qualities (Zhang et al., 2020). Hence, the reduction might be attributed to the degradation of tannin by microbial enzymes produced during fermentation (Dlamini et al., 2007). The enzyme called tannases and mono‐ and dioxygenases are capable of hydrolyzing complex tannins, hydrolysable tannins, and condensed tannins (Kuddus, 2018).

Trypsin inhibitors are protein inhibitors that limit the action of the enzyme trypsin (Cristina Oliveira de Lima et al., 2019). They inhibits the proteolytic enzyme trypsin that is secreted by the pancreas and thus affect the digestibility and bioavailability of protein (Sindhu & Khetarpaul, 2001). In the present study, trypsin inhibitor activity was decreased significantly (*p* < .05) after 48 h of fermentation with all fermentation setups. The highest reduction was observed for cocultures followed by *L. platurum* strain, *S. cerevisiae* strain, and naturally fermented flour. Similar observations have been reported by other researchers such as Ogodo et al., 2018; Adeyemo & Onilude, 2013; Osman & Gassem, 2013; Rahman & Osman, 2011 and Dordević et al., 2010. The reduction in trypsin inhibitor during fermentation might be attributed to microbial degradation of the trypsin inhibitor taking place throughout the fermentation process (Rahman & Osman, 2011).

Protein digestibility is a measure of the susceptibility of a protein to proteolysis and depends on the protein structure, thermal processing intensity, and presence of some compounds that are prejudicial to protein digestion, the so‐called antinutritional factors (Sá et al., 2019). It is also affected by other parameters such as pH, temperature, and ionic strength, all of which are directly related to proteolytic activities (Joye, 2019). In the present study, the protein digestibility of maize flour increased significantly (*p* < .05) at all fermentation setups after 48 h of fermentation. The highest increase was observed for *L. plantarum* strain followed by *S. cerevisiae* strain, cocultures and naturally fermented maize flour. This might be due to the fact that microorganisms such as lactic acid bacteria have the potential to produce proteolytic enzymes which could be responsible for increased protein digestibility (García‐Cano et al., 2019). Moreover, the reduction of antinutritional factors during fermentation indirectly increases the accessibility of proteins by enzymes and this in turn increases the protein digestibility. The present finding agrees with the finding of (Ogodo et al., 2018) who reported 61.28% to 88.70% increased protein digestibility of LAB consortium‐fermented maize flour.

## 4 CONCLUSION

This research showed that, fermentation with *L. plantarum*, *S. cerevisiae*, and their cocultures resulted in improved nutritional value of maize flour. Fermentation with these strains significantly increases the contents of protein and its digestibility of maize flour. However, fat, fiber, and carbohydrate contents were decreased. A significant reduction was also observed for phytate, tannin, and trypsin inhibitor activity of maize flour after fermentation with natural, *L. plantarum*, *S. cerevisiae* strains, and their cocultures. Fermented maize flour was higher in nutritional profiles compared to their unfermented counterparts due to activation of endogenous and exogenous enzymes that could be able to degrade antinutritional factors. Therefore, applications of these starter cultures in fermentation of maize flour at 37°C incubation temperature for 36 h are recommended for producing maize flour with enhanced nutritive value, which could be used to fight malnutrition.

## CONFLICT OF INTEREST

The authors declare that they have no conflict of interest.

## AUTHOR CONTRIBUTIONS

**Zemenu Kerie Terefe:** Conceptualization (lead); Data curation (lead); Formal analysis (lead); Funding acquisition (lead); Investigation (lead); Methodology (lead); Project administration (lead); Resources (lead); Software (lead); Validation (equal); Visualization (lead); Writing‐original draft (lead); Writing‐review & editing (lead). **Mary Nyambeki Omwamba:** Conceptualization (equal); Validation (equal); Visualization (equal); Writing‐review & editing (equal). **John Masani Nduko:** Conceptualization (equal); Methodology (equal); Validation (equal); Visualization (equal); Writing‐review & editing (equal).

## Data Availability

All the supporting data are available and will be provided up on request.
